# Degeneration of aflatoxin gene clusters in *Aspergillus flavus* from Africa and North America

**DOI:** 10.1186/s13568-016-0228-6

**Published:** 2016-08-31

**Authors:** Bishwo N. Adhikari, Ranajit Bandyopadhyay, Peter J. Cotty

**Affiliations:** 1 USDA-ARS, The University of Arizona, School of Plant Sciences, 303 Forbes Building, P.O. Box 210036, Tucson, AZ 85721 USA; 2 International Institute of Tropical Agriculture (IITA), PMB 5320, Oyo Road, Ibadan, Nigeria

**Keywords:** *Aspergillus flavus*, Aflatoxin gene cluster, Non-aflatoxigenic, Cluster degeneration, Biocontrol, Evolution

## Abstract

*Aspergillus flavus* is the most common causal agent of aflatoxin contamination of food and feed. However, aflatoxin-producing potential varies widely among *A. flavus* genotypes with many producing no aflatoxins. Some non-aflatoxigenic genotypes are used as biocontrol agents to prevent contamination. Aflatoxin biosynthesis genes are tightly clustered in a highly conserved order. Gene deletions and presence of single nucleotide polymorphisms (SNPs) in aflatoxin biosynthesis genes are often associated with *A. flavus* inability to produce aflatoxins. In order to identify mechanisms of non-aflatoxigenicity in non-aflatoxigenic genotypes of value in aflatoxin biocontrol, complete cluster sequences of 35 *A. flavus* genotypes from Africa and North America were analyzed. Inability of some genotypes to produce aflatoxin resulted from deletion of biosynthesis genes. In other genotypes, non-aflatoxigenicity originated from SNP formation. The process of degeneration differed across the gene cluster; genes involved in early biosynthesis stages were more likely to be deleted while genes involved in later stages displayed high frequencies of SNPs. Comparative analyses of aflatoxin gene clusters provides insight into the diversity of mechanisms of non-aflatoxigenicity in *A. flavus* genotypes used as biological control agents. The sequences provide resources for both diagnosis of non-aflatoxigenicity and monitoring of biocontrol genotypes during biopesticide manufacture and in the environment.

**Electronic supplementary material:**

The online version of this article (doi:10.1186/s13568-016-0228-6) contains supplementary material, which is available to authorized users.

## Introduction

*Aspergillus flavus*, the primary causal agent of food and feed contamination with the toxic fungal metabolites, aflatoxins (Cotty et al. 1994; Klich 2007; Probst et al. 2010, 2011), is ubiquitous in the environment. This prolific saprophyte (Klich 2002) is an opportunistic pathogen of plants and animals (Leger et al. 2000) including humans (Hedayati et al. 2007; Sepahvand et al. 2011). A wide variety of crops including maize, cottonseed, peanuts, and tree nuts are susceptible to infection and subsequent aflatoxin contamination (Cotty et al. 1994; Doster and Michailides 1994). Aflatoxin B_1_, the most toxic aflatoxin, is carcinogenic for both humans and animals (McKean et al. 2006). Aflatoxins in foods and feed are limited through strict regulation which results in significant economic loss for producers and processors of contaminated crops (Robens and Cardwell 2003; van Egmond et al. 2007).

Populations of *A. flavus* in agricultural fields are complex communities that contain many Vegetative Compatibility Groups (VCGs) (Cotty et al. 1994; Ehrlich et al. 2007). Aflatoxin-producing potential is known to vary less among genotypes within a VCG than among genotypes from different VCGs (Ehrlich and Cotty 2004) and the average aflatoxin-producing potential of populations varies among fields, areas, and regions (Cotty 1997; Lisker et al. 1993; Schroeder and Boller 1973). Endemic *A. flavus* genotypes that don’t produce aflatoxin, frequently called non-aflatoxigenic strains, have been used successfully as biocontrol agents to reduce aflatoxin contamination in cottonseed (Cotty 1989; Ehrlich et al. 2007), peanut (Dorner 2008, 2009; Dorner and Horn 2007), corn (Atehnkeng et al. 2008, 2014; Brown et al. 1999), and pistachio (Doster et al. 2014) where they competitively displace aflatoxin-producing fungi (Cotty and Mellon 2006). Biocontrol applications reshape the fungal community that grows in association with developing crops so that the non-aflatoxigenic genotypes dominate the fungal community structure and as a result the aflatoxin-producing potential of that fungal community is greatly reduced. Biological control products directed at reducing contamination and utilizing endemic well adapted, non-aflatoxigenic genotypes of *A. flavus* as active ingredients are registered for use in the US, Nigeria, and Kenya, and are under development in several other nations in Africa, the Americas, and Europe (Atehnkeng et al. 2014; Chulze et al. 2015; Cotty 2006; Mauro et al. 2013). The first non-aflatoxigenic genotype to be registered as a biopesticide active ingredient by a regulatory authority was *A. flavus* AF36 which, after more than a decade, is still used in commercial agriculture in the US on cottonseed, corn, pistachios, and figs (Grubisha and Cotty 2015).

Enzymes and regulatory proteins for aflatoxin synthesis in *A. flavus* are encoded by 25 clustered genes in a 70-kb region (Ehrlich et al. 2005; Yu et al. 2004b). The genes involved in aflatoxin biosynthesis are clustered together and the order of genes within the cluster is highly conserved within *Aspergillus* section *Flavi*. Presence and absence of a complete functional aflatoxin gene cluster is often associated with the ability to produce toxin by the members of the *Aspergillus* section *Flavi*. Deletion of portions of the aflatoxin biosynthetic gene cluster within non-aflatoxigenic *A. flavus* genotypes is common (Chang et al. 2005) and strains of *Aspergillus* section *Flavi* with large deletions in the aflatoxin gene cluster have been used to study the genetics of aflatoxin biosynthesis (Prieto et al. 1996). Both *A. sojae* and *A. oryzae*, close relatives of *A. flavus* which are used for food fermentation, don’t produce aflatoxins (Wei and Jong 1986) even though homologues of aflatoxin biosynthesis genes are present in the genomes of both species (Chang et al. 1995; Klich et al. 1995; Yu et al. 2000). Similarly, a single nucleotide polymorphism (SNP) in the aflatoxin pathway polyketide synthase gene in *A. flavus* AF36 (Ehrlich and Cotty 2004; Ehrlich et al. 2007) and deletion of the entire aflatoxin gene cluster (Chang et al. 2005; Dorner 2004) in *A. flavus* NRRL21882 (active ingredient of afla-guard®) are sufficient to explain non-aflatoxigenicity in these active ingredients of commercially used biopesticides. Nevertheless, mechanisms responsible for non-aflatoxigenicity are diverse and for most non-aflatoxigenic *A. flavus* genotypes, the specific genetic changes leading to non-aflatoxigenicity are unknown.

Degradation of the aflatoxin gene cluster in *A. oryzae* included indel and SNP mutations. Deletion of a large portion of the aflatoxin biosynthesis gene cluster, including *aflR*, was detected in 40 % of the *A. oryzae* RIB strains (groups 2 and 3) in analyses including 39 *A. oryzae* genotypes (Kusumoto et al. 2000). A number of mutations relative to the *A. flavus* sequence were observed in the aflatoxin biosynthesis gene cluster in *A. oryzae* RIB 40 (Tominaga et al. 2006). Eight deletion patterns were detected among 38 non-aflatoxigenic *A. flavus* genotypes from the southern United States (Chang et al. 2005). When selective forces necessary to maintain the aflatoxin gene cluster are relaxed, genetic drift may lead to further mutations and deletions in genes related to aflatoxin synthesis (Chang et al. 2005). Although the loss of production of aflatoxin by some non-aflatoxigenic genotypes is attributed to point mutations (Ehrlich and Cotty 2004) or small deletions in genes essential for aflatoxin production (Calvo et al. 2004), the process of degeneration of aflatoxin gene cluster is not well understood.

Non-aflatoxigenic *A. flavus* genotypes have been identified from different regions of the world (Cotty 1997; Joffe 1969; Lisker et al. 1993; Schroeder and Boller 1973) and abilities of these genotypes to reduce aflatoxin have been demonstrated in many crops (Probst et al. 2014). Previous studies on non-aflatoxigenic *A. flavus* have primarily focused on analysis of few genotypes through analysis of selected genes in the aflatoxin gene cluster and no study has compared complete genome sequences of aflatoxin gene clusters. Studies have primarily relied upon PCR amplification to evaluate gene presence (Callicott and Cotty 2015; Chang et al. 2005; Kusumoto et al. 2000; Tominaga et al. 2006). Such studies provide an incomplete picture of cluster structure and may result in misleading conclusions. Though the diversity of *A. flavus* biocontrol agents is associated with variation in the aflatoxin gene cluster and deletion is a major cause of non-aflatoxigenicity, the process of acquisition and the evolution of non-aflatoxigenicity needs more detailed description to facilitate both biopesticide regulatory processes and DNA based monitoring of non-aflatoxigenicity. Molecular elucidation of aflatoxin gene clusters in non-aflatoxigenic *A. flavus* also has important implications for understanding mechanisms of non-aflatoxigenicity and the evolution of aflatoxin gene clusters in *A. flavus*. Effectiveness of non-aflatoxigenic genotypes varies by crop, location and nutrient environment (Mehl and Cotty 2010, 2013; Mehl et al. 2012). Thus it is important to identify new genotypes that are well adapted to both target crops and target regions in order to optimally reduce aflatoxin concentration in infected crops. In order to identify mechanisms of non-aflatoxigenicity and understand the evolution of aflatoxin gene clusters in non-aflatoxigenic genotypes, we sequenced and analyzed aflatoxin gene clusters from 35 *A. flavus* genotypes from North America, West Africa and East Africa. By assembling the aflatoxin gene cluster and flanking regions, we analyzed the diversity of aflatoxin gene clusters of *A. flavus* genotypes currently incorporated as active ingredients in aflatoxin preventing biopesticides. The molecular characteristics of these factors will be useful for both understanding mechanisms of non-aflatoxigenicity and for monitoring non-aflatoxigenic genotypes on crops and in the environment.

## Materials and methods

### Fungal cultures

Thirty-five genotypes were chosen to represent the non-aflatoxigenic genotypes from Africa and North America. Non-aflatoxigenic genotypes of *A. flavus* from Kenya and Nigeria were collected and processed during previous studies (Donner et al. 2010; Probst et al. 2011). Genotypes from Burkina Faso and Senegal were genotyped and identified from samples transported to IITA’s plant pathology laboratory in Ibadan, Nigeria. Maize and soil samples collected in the United States (Texas and Arizona) were transported to the United States Department of Agriculture (USDA), Agricultural Research Service (ARS), at the University of Arizona. Genotype recovery, identification and aflatoxin quantification was done as previously (Cotty 1997; Cotty and Cardwell 1999). Non-aflatoxigenicity was confirmed on maize for all fungi from cultures resulting from two serial single spore transfers. Geographic origins and sources of the 35 genotypes are shown in Table 1.

**Table 1:** *Aspergillus flavus* genotypes used in the current study

	Country	Substrate	Group^a^	Genotype^b^	VCG	Culture accession/source^c^
East Africa	Kenya	Maize	A	C6-E	KN00A	USDA-ARS, Tucson, USA
			C	C8-F	KN012	USDA-ARS, Tucson, USA
			B	E63-I	KN001	USDA-ARS, Tucson, USA
			B	R7-H	KN011	USDA-ARS, Tucson, USA
West Africa	Burkina Faso	Groundnut	D	GO18-2	BF018	IITA, Ibadan, Nigeria
			A	GO67-10	BF067	IITA, Ibadan, Nigeria
		Maize	A	M011-8	BF011	IITA, Ibadan, Nigeria
			D	M092-15	BF092	IITA, Ibadan, Nigeria
			A	M102-11	BF102	IITA, Ibadan, Nigeria
			A	M109-2	BF109	IITA, Ibadan, Nigeria
			F	M110-7	BF110	IITA, Ibadan, Nigeria
			A	M129-5	BF129	IITA, Ibadan, Nigeria
	Nigeria	Maize	A	Ka16127	AV16127	IITA, Ibadan, Nigeria
			A	La3279	AV3279	IITA, Ibadan, Nigeria
			A	La3304	AV3304	IITA, Ibadan, Nigeria
			F	Og0222	AV0222	IITA, Ibadan, Nigeria
	Senegal	Maize	A	M2-7	SN002	IITA, Ibadan, Nigeria
			E	M21-11	SN021	IITA, Ibadan, Nigeria
			A	Ms14-19	SN014	IITA, Ibadan, Nigeria
		Sesame	A	Ss19-14	SN019	IITA, Ibadan, Nigeria
North America	USA	Cottonseed	A	AF36	YV36	NRRL 18543
		Maize	F	AT21-A	TX021	USDA-ARS, Tucson, USA
			F	AT4-C	TX004	USDA-ARS, Tucson, USA
			D	AT5-B	TX005	USDA-ARS, Tucson, USA
			A	BA16-F	TX016	FGSC A2220
			F	BA35-C	IC001	FGSC A2223
			A	BY18-A	EC11-D	USDA-ARS, Tucson, USA
			F	BY19-D	IC001	USDA-ARS, Tucson, USA
			A	DO107-L	TX107	USDA-ARS, Tucson, USA
			D	DO114-A	TX114	USDA-ARS, Tucson, USA
			C	DO38-B	TX038	FGSC A2226
			F	DO46-G	TX046	FGSC A2229
			F	EC19-B	TX019	USDA-ARS, Tucson, USA
			A	EC69-E	EC69-E	USDA-ARS, Tucson, USA
			F	NRRL21882		NRRL21882
		Cottonseed		AF13	YV13	ATCC 96044
				EB01	EB01	USDA-ARS, Tucson, USA
				MR2-17	MR17	USDA-ARS, Tucson, USA
				OD02	OD02	USDA-ARS, Tucson, USA

^a^Refers to the grouping based on aflatoxin gene cluster alignment in Fig. 1 and Neighbor-Net network in Fig. 2

^b^Isolates from Kenya were first reported in Probst et al. (2011); isolates from Nigeria were first reported in Atehnkeng et al. (2008); AF13 and AF36 were first reported in Cotty (1989); EB01 and MR2-17 were first reported in Mehl and Cotty (2013); and OD02 was first reported in Grubisha and Cotty (2009). Rest of the isolates are first reported in this manuscript

^c^Culture collection/source designation (IITA, The International Institute of Tropical Agriculture, Oyo Road, Ibadan, Nigeria; NRRL, ARS Culture Collection; FGSC, Fungal Genetics Stock Center)

### DNA isolation, library preparation and sequencing

Genomic DNA was isolated from conidia collected after 7 days culture (31 **°**C, dark) on 5 % V-8 vegetable juice, 2 % salt, and 2 % agar. The FastDNA SPIN Kit and the FastPrep Instrument were used following the manufacturer’s instructions (MP Biomedicals LLC, Santa Ana, CA, USA). DNA from the FastDNA SPIN Kit was applied to a SPIN filter column following manufacturers instructions to remove small DNA fragments and other contaminants <10,000 Da. Genomic DNA was quantified with both spectrophotometer (modelND-1000, NanoDrop) and with the Qubit dsDNA BR assay kit (Q32850) using the Qubit 1.0 Fluorometer (Thermo Fisher Scientific, Waltham, MA) following the manufacturer’s guidelines. Libraries were prepared according to Illumina’s HiSeq 2000 library preparation protocol (Illumina, San Diego, CA, USA). Whole genome sequencing was performed at Arizona Genomics Institute (AGI) located at the University of Arizona’s BIO5 institute using Illumina HiSeq 2000. Libraries were sequenced with 100-bp paired-end reads and an insert size of 250-bp.

### Aflatoxin gene cluster assembly and comparative analysis

To ensure the quality of the reads, the raw reads obtained from AGI were quality trimmed and cleaned using cutadapt v1.8.3 (Martin 2011). Cleaned reads were assembled using a de novo genome assembly program Velvet v1.2.10 (Zerbino and Birney 2008). The aflatoxin gene cluster from each assembled genome was extracted using the published aflatoxin gene cluster of *A. flavus* AF13 as a reference (Ehrlich et al. 2005) through BLAST alignment. Aflatoxin gene clusters were annotated using MAKER (Cantarel et al. 2008) genome annotation pipeline and multiple alignments were performed using CLUSTALW (Thompson et al. 1994) and MUMMER (Delcher et al. 2002). Putative functional annotation was assigned by searching the gene models against UniProt (Bateman et al. 2015) and previously annotated aflatoxin gene clusters from *A. flavus* using BLASTX (Altschul et al. 1990). To investigate the evolution of several portions of the aflatoxin-gene cluster, we divided the aflatoxin gene cluster pathway into two halves considering *ver*-*1* as a midpoint (Roze et al. 2007) and thus excluded from the analysis when comparing early and late portions of the gene cluster. The early part of the cluster includes *norB* through *norA* genes on the telomere side of the cluster and later part includes *verA* through *hypA* genes on the centromere side of the cluster. The assembled aflatoxin gene clusters are deposited at EMBL-EBI (ENA) database under the study accession number PRJEB11911.

### Variant analysis

Single nucleotide polymorphisms (SNPs) in aflatoxin gene clusters were computationally detected using two SNP calling methods. First, reads from different genotypes were mapped to aflatoxin gene cluster from *A. flavus* AF13 using Bowtie v1.1.1 (Langmead et al. 2009) and the resulting BAM file was fed to SAMTools v0.1.16 (Li et al. 2009). SNPs positions were identified using mpileup function in SAMTools. SNPs were filtered using vcfutils.pl varFilter with minimum mapping quality (-Q) of 20 and minimum and maximum read coverage of 20 and 100 respectively. Second, to improve the confidence in variant calling, SNPs were also identified using MAQ v0.7.1 (Li et al. 2008) easyrun pipeline. SNPs were filtered using maq.pl SNPfilter with minimum read depth (-d) of 20, minimum consensus quality (-q) of 20, and minimum adjacent consensus quality (-n) of 20. Only SNPs called by both methods were considered for the analysis. In order to find the false positive rate of SNP called by two different methods used in this analysis, a random sample of 3–9 SNPs from six different genes were selected for validation. SNP validation was done by manually checking the alignment and by PCR amplification and sequencing of the polymorphic sites in five genotypes. Deletions in aflatoxin gene clusters were predicted using variant detection program DELLY (Rausch et al. 2012). By using paired-end mapping and split-read analysis, DELLY identifies deletions, duplications, inversions and translocations in the genomes. Deletion predictions with supporting reads less than three and the mapping quality below 20 were rejected. Only deletions greater than 10 bp were considered for further analysis. Deletions predicted by DELLY were validated by manually checking the alignments and through PCR, by designing primers that bridge the putative gaps or that amplify from regions flanking the gap within the deleted region. PCR conditions used to validate the deletions were described previously (Mehl and Cotty 2010).

### Neighbor-Net network

Genetic relationships among the 35 genotypes were examined by using simple sequence repeat (SSR) data for 17 loci identified in the previous study (Grubisha and Cotty 2009) scored manually for all included genotypes according to their amplified fragment size. Genetic distance was calculated across 17 loci with START2 program (Jolley et al. 2001). The distance matrix was analyzed with the Neighbor-Net algorithm in SplitsTree v4.13.1 (Huson and Bryant 2006). The tree was displayed using SplitsTree. Edges were color shaded according to the grouping of genotypes based on structure of aflatoxin gene cluster.

### Identification of similarly evolving genes

To identify the genes evolving together, correlations between SNP densities from different genotypes were calculated. Pearson’s correlation coefficient, *r*, was calculated for every gene combination in the combined aflatoxin biosynthesis and sugar clusters. Groups of four genes each from early and late portions of the aflatoxin gene cluster and from the sugar cluster were selected and the level of correlation of these gene groups with each gene in both clusters was estimated as the mean of the correlation coefficients of the four groups of genes with that gene. Two or more genes were considered evolving together if the average correlation coefficient between them was higher than 0.50 and considered highly evolving if correlation coefficient was higher than 0.70 (Fraser et al. 2004).

### Ka/Ks analysis

To measure the rates of evolution of aflatoxin gene cluster genes, the ratio of non-synonymous to synonymous substitutions (*Ka/Ks*) between pairs of genes from five non-aflatoxigenic genotypes and toxigenic genotype *A. flavus* AF13 was calculated using the yn00 package in PAML (Yang 2007). Five non-aflatoxigenic genotypes were selected to represent the genotypes with full and partial aflatoxin gene clusters. Four to six genes representing early, middle and late segments of the aflatoxin gene cluster were examined from genotypes with complete as well as partial gene clusters. Only orthologous genes were used for the analysis.

### Statistical analysis

The number and density of SNPs were subjected to analysis of variance (ANOVA). Pearson’s correlation coefficient was calculated on SNP densities. Tests of differences in means were performed following significant ANOVAs with Tukey’s honest significant difference (HSD) test. Pair-wise comparisons of number of synonymous to non-synonymous SNPs, SNP densities and *Ka/Ks* values were done using Paired t test. Statistical analyses were performed with SAS (version 9.1, SAS Institute Inc., Cary, NC, USA).

## Results

### Variation in the aflatoxin gene cluster

Whole genome sequence of the non-aflatoxigenic genotypes analyzed in this study (Table 1) was obtained by aligning assembled genomes with published aflatoxin gene cluster from *A. flavus* AF13 (Ehrlich et al. 2005). For all genotypes, the aflatoxin gene cluster was part of a large contig/scaffold, which mapped to the sub-telomeric region of chromosome 3 of *A. oryzae* RIB40 (Machida et al. 2005). Distribution of genes within the cluster in *A. oryzae* RIB40 and directional alignment of each are identical in *A. flavus* NRRL3357 (Ehrlich et al. 2005) and *A. parasiticus* SRRC 2043 (Yu et al. 2004a).

**Fig. 1:**
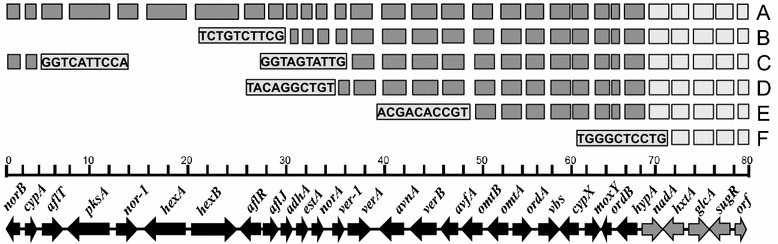
Schematic of the aflatoxin biosynthesis (*dark grey*) and sugar clusters (*light grey*) from 35 non-aflatoxigenic *A. flavus* genotypes. The *bottom figure* shows the aflatoxin gene cluster from *A. flavus* AF13 (Ehrlich et al. 2005) and genes putatively involved in sugar translocation. Genotypes are grouped based on presence or absence of genes. Absence of genes towards the telomeric end of the cluster indicates gene deletion. Sequences bordering deletions are indicated in *grey shaded boxes*. *Letters* on the *right* indicate groups of genotypes with similar clusters. Group A includes AF36, BA16-F, BY18-A, C6-E, DO107-L, EC69-E, GO67-10, Ka16127, La3279, La3304, M011-8, M102-11, M109-2, M129-5, M2-7, Ms14-19, Ss19-14; group B includes E63-I and R7-H; group C includes C8-F and DO38-B; group D includes AT5-B, DO114-A, GO18-2 and M092-15; group E includes M21-11; group F includes AT21-A, AT4-C, BA35-C, BY19-D, DO46-G, EC19-B, M110-7, NRRL21882, and Og0222

**Fig. 2:**
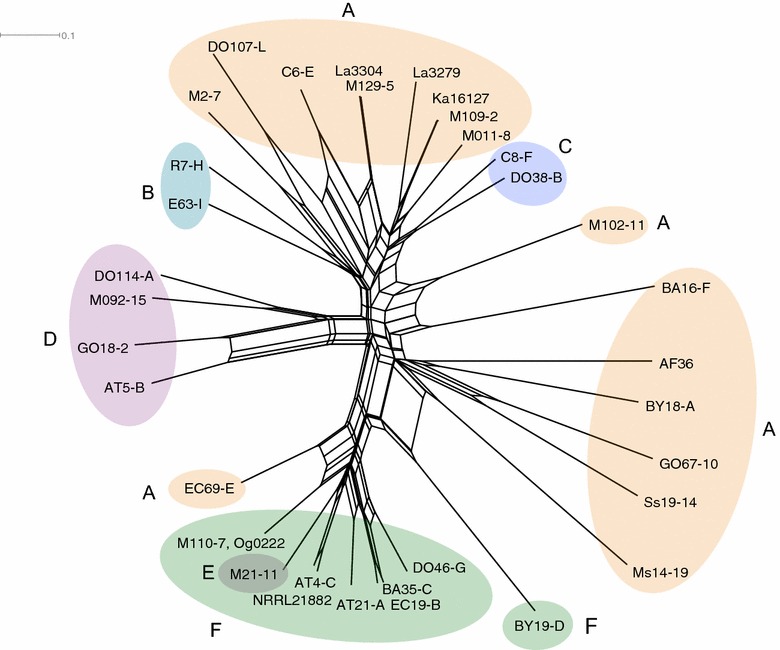
Neighbor-Net network of 35 non-aflatoxigenic *A. flavus* genotypes based on 17 SSR loci. Network was generated by the split decomposition algorithm with the distance matrix calculated by START2 from allelic profile data using SplitsTree 4 (Huson and Bryant 2006). Nodes are colored *orange* (complete cluster), *purple* and *teal* (partial deletion) and *green* (complete deletion) based on completeness of the aflatoxin gene cluster. The *letters* correspond with the grouping of genotypes based on aflatoxin gene cluster sequence alignment and detailed under the “Variation in the aflatoxin gene cluster” section of the results

Analyses of aflatoxin gene clusters from 35 genotypes showed high level of diversity in terms of number and size of deletions in the genes (Fig. 1). Based on the size and type of deletion, genotypes ranged from those with full aflatoxin gene cluster to those having no genes at all with most deletions happening at the left end of the cluster or towards the telomeric end (Fig. 1). Based on the structure and presence of genes, genotypes were divided into 6 different groups (Group A through F). Group A contains 17 genotypes (49 % of examined genotypes) including AF36, a commercially used biocontrol agent, that have all the aflatoxin cluster genes. Groups B through E contain partial aflatoxin gene clusters with varying level of deletions and Group F contains genotypes with none of the aflatoxin genes. Group B contains two genotypes (6 % of examined genotypes) with an identical deletion including 11 genes. In addition to those 11 genes, genotypes in this group have also lost part of *aflJ* gene. The terminal gene, *aflJ*, from these two genotypes has an identical deletion indicating a shared deletion event. Group C, includes two genotypes that share identical deletions at the left end of the cluster. Genotypes in this group contain two genes (*norB* and *cypA*) on the left end and lost 13 genes leaving partially deleted *verA* at the middle of the cluster. Similarly, group D contains four genotypes (12 % of examined genotypes) with deletion of 14 genes including *norA* but with *ver*-*1* left intact. Group E contains only one genotype with a large deletion including 19 complete genes and partial deletion of *omtB* gene. Despite the presence of partial gene clusters in groups B, C, D and E each group has remnants of different deletion events as indicated by the sequenced ends (Fig. 2). The fact that groups B, C, D and E have varying level of deletions suggest each went through different deletion events. Finally, Group F contains a distinct set of nine biocontrol genotypes (27 % of examined genotypes) including NRRL21882 with a completely deleted aflatoxin gene cluster. In addition to genes from the aflatoxin gene cluster, *nadA* gene from sugar cluster is also deleted from this group.

### Evolutionary relationship between *A. flavus* genotypes

In order to examine the genetic relationships among non-aflatoxigenic genotypes analyzed in this study, a Neighbor-Net network (Fig. 2) was constructed using the genetic distance estimated from SSR data from 17 loci distributed throughout 8 chromosomes of *A. flavus* (Grubisha and Cotty 2009). The Neighbor-Net network has a high degree of congruence with the genotype graphs (group A–F) revealed by sequencing (Figs. 1, 2). The nine genotypes in group F (lacking the entire aflatoxin gene cluster) are clustered together in the same region of the Neighbor-Net network. Nested within group F is the sole genotype in group E with over 19 genes deleted. The Neighbor-Net network clustered all isolates by group for groups B, C, and D. The only group for which genotypes were not consistently grouped together was group A, the ancestral genotype. Genotypes in group A contain all the aflatoxin biosynthesis genes, each with distinct aspects of degeneration partially described by the lack of shared deletion pattern (Table 2). These observations are consistent with groups B through F arising from group A, the ancestral state. The network represents a nontree-like structure that recapitulates diversity but fails to show clustering based on geographic origins. Despite having different levels of partial deletion of aflatoxin genes, groups B, C, D and E are not clustered together (Fig. 2). Among the genotypes, genotypes with similar sequence ends are in the same group suggesting distinct deletion events for each group.

**Table 2:** Small (<1 kb) deletions in aflatoxin biosynthesis genes sequenced from 26 genotypes of *Aspergillus flavus*

Genotype	Group	No of deletions	*cypA*	*aflT*	*pksA*	*hexA*	*aflR*	*verA*	*verB*	*avfA*	*omtB*	*vbs*	*cypX*	*ordB*
AF36*	A	4	*17* ^a^		*61*		*29*						*21*	
AT5-B	D	2							*57*					*33*
BA16-F*	A	6	*17*	*253*	*61*			*20*	*57*				*21*	
BY18-A*	A	6	*17*		*61*	*278*	*29*	*20*		*29*				
C6-E*	A	6	*23*		*61*		*29*	*20*				*42*	*21*	
DO107-L*	A	4	*23*		*61*		*29*			*29*				
DO114-A	D	2							*57*					*33*
DO38-B	C	2	*17*											*33*
C8-F	C	2	*17*											*33*
E63-I	B	3							*57*	*29*	*25*			
EC69-E*	A	4	*17*	*42*	*61*									*33*
GO18-2	D	2							*57*					*33*
GO67-10*	A	4	*23*		*61*			*20*		*29*				
Ka16127*	A	6	*23*		*61*	*278*	*29*	*20*		*29*				
La3279*	A	9	*17*		*61*	*278*	*29*	*20*	*57*	*29*		*42*	*21*	
La3304*	A	5	*17*		*61*	*278*	*29*			*29*				
M011-8*	A	8	*17*		*61*	*278*	*29*	*20*		*29*		*42*	*21*	
M092-15	D	1							*57*					
M109-2*	A	6	*17*		*61*	*278*	*29*			*29*				*33*
M129-5*	A	6	*17*		*61*	*278*	*29*	*20*		*29*				
M2-7	A	7	*17*		*61*	*278*	*29*	*20*		*29*			*21*	
Ms14-19*	A	7	*17*	*253*	*61*			*20*	*57*				*21*	*33*
R7-H	B	3							*57*	*29*	*25*			
Ss19-14*	A	7	*17*	*253*	*61*			*20*	*57*				*21*	*33*
M102-11	A	5	*23*		*61*		*29*	*20*		*29*				
M21-11	E	1											*21*	
		Total deletions	19	4	17	8	12	12	10	13	2	3	9	9

^a^ Numbers in italics indicate deletion sizes in bp

*These genotypes have complete sets of genes in aflatoxin gene cluster

### Deletion in aflatoxin gene cluster

In addition to deletions in the *norB*–*cypA* region present in all *A. flavus* (Chang et al. 2005; Probst et al. 2014), several other large deletions in the aflatoxin-biosynthesis gene cluster have been described (Callicott and Cotty 2015; Chang et al. 2005; Donner et al. 2010). The current study revealed both large (>1-kb, Fig. 1) and small (Table 2; Additional file 1: Table S1) deletions through alignment of sequences of 35 non-aflatoxigenic genotypes with the sequence of *A. flavus* AF13 (Ehrlich et al. 2005). Deletions were widespread across the clusters (Table 2; Fig. 1). Group A has deletions ranging from 17–278 bp (Table 2; Additional file 1: Table S1). Although deletions were found in 12 of the 25 genes present in the cluster; *cypA*, *pksA*, *aflR*, *verA*, *verb* and *avfA* genes have deletions in at least ten genotypes analyzed. Genotypes in Group B share the same approximately 30-kb deletion on the telomeric end of the cluster with partial *aflJ* gene remaining at the end of the cluster. Group C genotypes have unique approximately 40-kb deletion with *norB* and *cypA* genes at the telomeric end of the cluster. Genotypes in Group D have an identical approximately 35-kb deletion. Group E genotype has approximately 50-kb deletion in the aflatoxin gene cluster. Group F is composed of genotypes that lack all genes in the aflatoxin gene cluster. Genotypes in the same group (Group B through F) have identical sequence ends, indicating that the deletion event in each group probably occurred in a common ancestor. In addition to larger deletions, genotypes in group A, B, C, D, and E have 1-9 smaller deletions of varying sizes scattered among 12 genes (Table 2; Additional file 1: Table S1). Of the 35 genotypes analyzed in this study, more than 49 % have larger deletions in between *norB* to *estA* (Table 3) whereas smaller deletions were more concentrated in *norB*, *cypA*, *pksA, aflR, verA* followed by *verB*. The results from validation clearly showed that the smaller deletions were real (Additional file 1: Table S1). In line with the deletion pattern, it is interesting to note that the genes in early portions of the cluster are present only in 49–69 % of genotypes while genes in late portions of the cluster are present in at least 71–74 % of the genotypes (Table 3). Four genes from the sugar cluster (*hxtA*, *glcA*, *sugR* and *orf*) were the only genes present in all genotypes.

**Table 3:** Frequencies of aflatoxin biosynthesis and sugar cluster genes among 35 atoxigenic genotypes from Africa and North America

Cluster^a^	Gene^b^	Genotype (%)^c^	Synonymous SNPs^d^	Non-synonymous SNPs^d^	Nonsense SNPs^e^
Early genes	*norB*	19 (54)	0–3 (43)	0–2 (49)	0
	*cypA*	19 (54)	0–3 (43)	0–5 (43)	0–1 (49)
	*aflT*	17 (49)	0–3 (43)	0–4 (43)	0
	*pksA*	17 (49)	6–18 (40)	3–16 (40)	0–1 (3)^f^
	*nor*-*1*	17 (49)	0–1 (6)	0–1 (3)	0
	*hexA*	17 (49)	5–18 (40)	2–15 (40)	0
	*hexB*	17 (49)	9–20 (49)	7–15 (49)	0
	*aflR*	17 (49)	1–3 (49)	1–3 (49)	0
	*aflJ*	19 (54)	2–9 (51)	0–2 (51)	0–1 (3)
	*adhA*	19 (54)	1–2 (54)	1–4 (49)	0
	*estA*	19 (54)	2–6 (54)	1–3 (54)	0
	*norA*	24 (69)	0–5 (46)	0–3 (46)	0–1 (3)
	***ver*** **-** ***1***	25 (71)	0–5 (63)	0–2 (57)	0
Late genes	*verA*	25 (71)	0–37 (69)	1–19 (71)	0
	*avnA*	27 (77)	2–5 (77)	2–5 (77)	0
	*verB*	25 (71)	0–5 (69)	1–3 (71)	0
	*avfA*	25 (71)	0–4 (57)	0–4 (63)	0
	*omtB*	26 (74)	0–6 (57)	1–3 (63)	0
	*omtA*	26 (74)	0–7 (66)	0–4 (71)	0–1 (3)
	*ordA*	26 (74)	0–9 (63)	0–6 (57)	0
	*vbs*	26 (74)	1–12 (74)	1–6 (74)	0–1 (3)
	*cypX*	26 (74)	1–11 (74)	1–3 (74)	0
	*moxY*	26 (74)	0–7 (69)	1–8 (74)	0
	*ordB*	26 (74)	0–2 (69)	0–2 (66)	0–1 (14)
	*hypA*	26 (74)	2–5 (74)	1–6 (74)	0–1 (14)
Sugar cluster	*nada*	26 (74)	1–2 (74)	1–6 (74)	0–3 (63)
	*hxtA*	35 (100)	0–5 (89)	1–2 (100)	0
	*glcA*	35 (100)	1–3 (100)	2–3 (100)	0
	*sugR*	35 (100)	1–5 (100)	0–4 (77)	0–1 (14)
	*orf*	35 (100)	0–3 (83)	1–2 (100)	0

^a^Classification is based on the enzymes encoded by these genes, which are involved in early and late portions of aflatoxin biosynthesis, considering *ver*-*1* (bold italics) as the middle gene

^b^Genes are listed in the same order as in aflatoxin gene cluster

^c^Percentages were calculated as (number of genotypes with the gene or SNP/total number of genotypes) × 100. The total number of genotypes was 35

^d^Number of synonymous or non-synonymouse SNPs present. Numbers in the parenthesis include percentage of isolates with at least one synonymous or non-synonymous SNP

^e^Number of nonsense SNPs and percentage of isolate with at least one nonesense SNP

^f^Stop codon present in *A. flavus* AF36 as reported in Ehrlich and Cotty (2004)

### Polymorphism in aflatoxin gene cluster

**Fig. 3:**
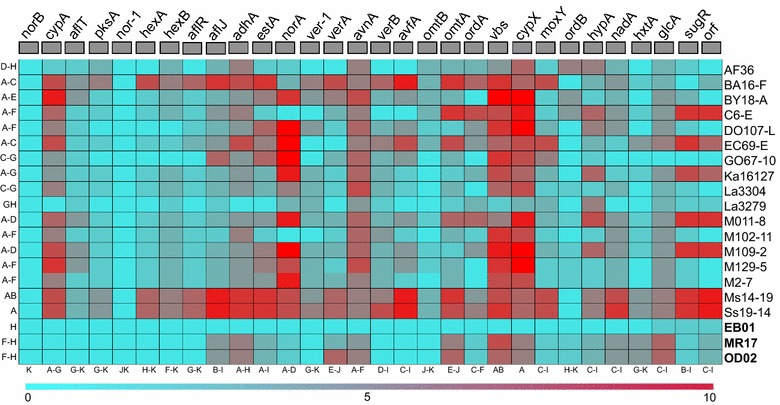
Heat map of SNP density (SNPs/kb of gene) in aflatoxin gene cluster of 17 non-aflatoxigenic and 3 toxigenic *A. flavus* genotypes. SNPs were called in reference to *A. flavus* AF13. Genotypes with a common *letter* along the *bottom* and genes with a common *letter* along the *side* do not differ significantly in mean SNP density by Tukey’s HSD test (*P* < 0.05). Only genotypes having all the genes in the aflatoxin biosynthesis cluster are included. Genotype names in *bold* are toxigenic. Schematic representation of the aflatoxin biosynthesis cluster is presented at the *top*. *Left* is the telomeric end of the cluster

Single nucleotide polymorphisms (SNPs) were identified by mapping Illumina sequencing reads from different genotypes to the *A. flavus* AF13 aflatoxin gene cluster (Ehrlich et al. 2005). Analysis included 26 non-aflatoxigenics with complete (17 genotypes) and partial (9 genotypes) sets of aflatoxin genes and 3 toxigenic genotypes. The average SNP density (SNPs/kb of gene) for non-aflatoxigenic and toxigenic genotypes varied from 2–6 and 0–2 respectively (Fig. 3). Although the average SNP density for all genotypes was 4 SNPs/kb, non-aflatoxigenic genotypes have significantly higher SNP density (4) as compared to toxigenic genotypes (2) (*P* < 0.05). Among 25 genes in aflatoxin gene cluster, *nor*-*1* has the lowest average SNP density (less than 1) while *omtA* has the highest (8) followed by *vbs* (7) and *cypX* (7) (Fig. 3). For the genotypes with partial aflatoxin gene cluster, the average SNP density for genes ranged from 0–6 SNPs/kb but average SNP density for genotypes ranged from 1–7 SNPs/kb. Of all SNPs, we found transitions to be over-represented: 64–74 % of all single base pair substitutions were transitions. Examination of polymorphic sites from aflatoxin biosynthetic gene cluster showed all analyzed sites with true polymorphisms. The results from validation clearly showed that the SNP calling methods are robust and selected SNPs, which were called by two methods, have very low false positive rate (Additional file 1: Table S2). Genotypes from the same geographical region were not necessarily similar in terms of number and density of SNPs. For example, the four genotypes from Senegal were no more similar to each other than to the genotypes from North America; nevertheless the genotypes with similar deletion patterns have more shared SNPs than genotypes with different deletions (Fig. 3).

To further investigate SNPs across the cluster, annotation was performed with Annovar (Wang et al. 2010). The effect of SNP mutation varied among genotypes. Synonymous SNPs were significantly more abundant (*P* < 0.005) than non-synonymous SNPs for all genotypes analyzed. Of the 25 aflatoxin cluster genes, eight genes (*cypA, pksA, aflJ, norA, omtA, vbs, ordB, hypA*) have stop-gain SNPs, four (*norB, cypA, ordB, glcA*) have stop-lost SNPs, and one gene (*hexA*) has a start-lost SNP eliminating a start codon (Table 3; Additional file 1: Table S3). To better understand SNP distribution, we analyzed SNP density in early and late portions of the gene cluster. The later portions had significantly higher SNP density (*P* < 0.05) (Fig. 3).

### Selective pressure in aflatoxin gene cluster

**Fig. 4:**
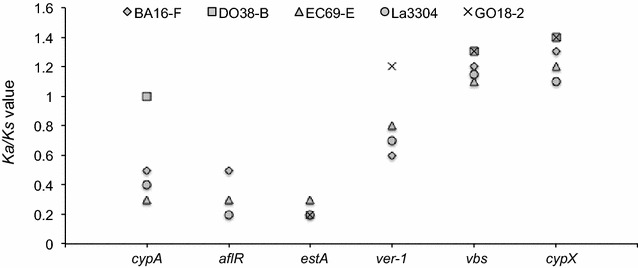
Plot of *Ka/Ks* values on *Y-axis* based on pairwise comparisons of orthologous genes from non-aflatoxigenic genotypes with *A. flavus* AF13. The length of coding sequence used to calculate *Ka/Ks* are as follow: *cypA* (390 bp), *aflR* (334 bp), *estA* (345 bp), *ver*-*1* (389 bp), *vbs* (332 bp), and *cypX* (327 bp). Since not all genes are present in all genotypes, no values are present for the *aflR* and *estA* genes in DO38-B and for the *cypA* and *aflR* genes in GO18-2. Genotypes BA16-F, EC69-E and La3304 have complete sets of genes while genotypes DO38-B and GO18-2 have partially deleted aflatoxin gene clusters

To study the evolution of the genes within the aflatoxin gene cluster, *Ka/Ks* ratio was calculated for pairs of orthologous genes between non-aflatoxigenic genotypes and toxigenic *A. flavus* AF13. Six genes representing early, middle and late portions of the cluster were selected from five genotypes either with full or partial sets of aflatoxin genes (Fig. 4). Of the six genes analyzed in this study, *cypX* and *vbs* have *Ka/Ks* > 1 for all genotypes, possibly indicating positive selection acting on these genes. The two genes, which are not present in all genotypes, with lowest *Ka/Ks* ratio were *aflR* and *estA*. Out of six genes, *aflR*, *cypX, estA* and *vbs* have *Ka/Ks* ratio either higher or lower than 1 for all genotypes analyzed. On the contrary, *cypA* and *ver*-*1* have a higher *Ka/Ks* ratio only for genotypes with partial gene cluster and lower for all genotypes with full gene clusters, suggesting that these two genes are evolving differently in different genotypes. Results showed that *cypX* and *vbs* have significantly higher *Ka/Ks* values (*Ka/Ks* > 1, *P* < 0.05, *t test*) than *aflR* and *estA* while *cypA* and *ver*-*1* are intermediate to those two groups of genes.

### Differential evolution of genes

In order to identify the genes that are evolving together, correlation coefficients were calculated for SNP densities from 25 aflatoxin genes and 5 genes in sugar cluster from 17 non-aflatoxigenic genotypes with complete gene sets. In order to represent the whole cluster, two groups of four genes from the aflatoxin gene cluster, representing early and late portions, and one group from the sugar cluster were selected. The two groups from the aflatoxin gene cluster were designated as the *pksA* group (containing *pksA*, *nor*-*1*, *hexA* and *hexB* genes) and the *cypX* group (containing *ordA*, *vbs*, *cypX*, *moxY* genes) while the group from the sugar cluster was designated as the *sugR* group (containing *hxtA*, *glcA*, *sugR*, *orf* genes) (Table 4). Because it has been shown that one of the genes from sugar cluster (*hxtA*) is concurrently expressed with aflatoxin genes (Yu et al. 2000), we reasoned that genes within these clusters might be evolving together. Considering Pearson’s correlation coefficient threshold of 0.5, all four genes within *pksA* group were highly evolving together (Pearson’s r = 0.85) but no such correlation was found with the *cypX* (Pearson’s r = 0.21) and *sugR* groups (Pearson’s r = 0.19) (Table 4). In contrast, genes from the *cypX* group were not evolving with genes from either *cypX* (average r = 0.35), *pksA* (average r = 0.21) or *sugR* (Pearson’s r = 0.15) groups. Four out of five genes from the sugar cluster, which are included in the *sugR* group, were evolving together (Pearson’s r = 0.66) while there was complete lack of such relationship with genes from the *pksA* (Pearson’s r = 0.19) and *cypX* (Pearson’s r = 0.15) groups.

**Table 4:** Correlations among SNP densities in three regions of the aflatoxin-biosynthesis and sugar clusters

Group	Gene	SNP density	Significance	*pksA* group^a^	Significance^b^	*cypX* group	Significance	*sugR* group	Significance
*sugR* group	*hxtA*	2		0.19	e	0.35	cde	0.7	ab
	*glcA*	4		0.15	e	0.01	e	0.55	a
	*sugR*	5		0.22	e	0.05	e	0.7	ab
	*Orf*	4		0.22	e	0.19	e	0.71	ab
	Average	4	AB	0.19	CD	0.15	D	0.66	B
*cypX* group	*ordA*	3		0.19	e	0.31	cde	0.35	cde
	*vbs*	7		0.27	de	0.34	cde	0.01	e
	*cypX*	8		0.27	de	0.41	bcd	0.05	e
	*moxY*	4		0.12	e	0.36	cde	0.19	e
	Average	6	A	0.21	CD	0.35	C	0.15	D
*pksA* group	*pksA*	2		0.87	a	0.19	e	0.19	e
	*nor*-*1*	0		0.83	a	0.27	de	0.15	e
	*hexA*	2		0.9	a	0.27	de	0.22	e
	*hexB*	3		0.82	a	0.12	e	0.22	e
	Average	2	B	0.85	A	0.21	CD	0.19	CD

^a^Values are means of either 3 (within group comparisons) or 4 (between group comparisons) correlation coefficients. Self-correlations (only occur in within groups comparisons) are not included to avoid bias

^b^Means followed by the same letter do not differ significantly (*P* < 0.05) by Tukey’s HSD test

## Discussion

Genotypes of *A. flavus* that do not produce aflatoxins exist in all warm agricultural areas (Cotty et al. 1994; Probst et al. 2011). These non-aflatoxigenics are used to reduce aflatoxin contamination of maize, cottonseed, groundnut, pistachio, and fig (Bandyopadhyay and Cotty 2013; Dorner 2008; Doster et al. 2008, 2014; Probst et al. 2014). Applications of non-aflatoxigenics reduce aflatoxins by altering compositions of fungal populations associated with crops (Antilla and Cotty 2002; Dorner 2004). As frequencies of non-aflatoxigenics increase, quantities of aflatoxins in the crop decreases (Cotty 1994). Non-aflatoxigenicity in *A. flavus* genotypes originate from deletions of aflatoxin-biosynthesis genes, including deletion of the entire cluster as in NRRL 21882 the active ingredient of afla-guard™ (NRRL 21882), or from a single inactivating SNP, as with the SNP induced stop codon in the *pks* gene of the biopesticide *A. flavus* AF36 (Ehrlich and Cotty 2004; Chang et al. 2005). However, processes through which aflatoxin-biosynthesis gene clusters of non-aflatoxigenics evolve and mechanisms behind cluster degeneration have not been adequately described for many biopesticide active ingredients. By sequencing and comparatively analyzing complete aflatoxin biosynthesis gene clusters of 35 non-aflatoxigenic genotypes of interest as active ingredients of biopesticides for aflatoxin mitigation in Africa and North America, the current work sheds light on the process of cluster degeneration. The results suggest the cluster is not evolving as one unit but that portions are responding divergently to selective forces and the degeneration process is not similar for all non-aflatoxigenic genotypes.

### Diversity of non-aflatoxigenic genotypes

Analyses of complete aflatoxin gene cluster sequences from 35 non-aflatoxigenic *A. flavus* genotypes from Africa and North America suggest 6 genotype groups based on cluster structure (Figs. 1, 2). Group A genotypes retain all aflatoxin biosynthesis genes and include the active ingredient of the USEPA registered biocontrol product *A. flavus* AF36 (AF36, Antilla and Cotty 2002). Group A genotypes have small (<1 kb) deletions (Table 2) and SNPs with potential to cause non-aflatoxigenicity. Frequently single genotypes have multiple genomic lesions sufficient to individually cause non-aflatoxigenicity suggesting phenotypic stability and long histories of the non-aflatoxigenicity. Groups B, C, D, and E consist of genotypes with varying levels of deletion, ranging from a few bases to most of the cluster. Group F is distinguished by absence of all aflatoxin biosynthesis genes resulting from a single group-wide deletion. Although deletions in aflatoxin gene clusters are known for *A. flavus* (Chang et al. 2005) and *A. oryzae* (Kusumoto et al. 2000), in the current study 35 genotypes were assembled against a reference sequence from an aflatoxin producer to reveal previously unknown diversity in both deletion pattern and mechanism of non-aflatoxigenicity. Genotypes within the same group have identical deletion patterns, including identical sequences bordering the deletions, suggesting common ancestry. Common ancestry is supported by the Neighbor-Net network based on 17 SSR loci (Grubisha and Cotty 2009) (Fig. 2). Clustering occurs among genotypes with similar deletion patterns but no clustering is seen based on genotype geographic origin reflecting both shared ancestry within each group and migration of groups across vast landscapes during intragroup divergence and after arisal of atoxigenicity.

### Degeneration of aflatoxin gene cluster

A variety of mutations shape genes and genomes. Protein coding genes may be eliminated by large deletion and smaller deletions may render genes inactive. In agreement with previous reports (Callicott and Cotty 2015; Chang et al. 2005; Criseo et al. 2001; Mauro et al. 2013; Yin et al. 2009), our results provide strong evidence that partial or complete gene deletion is an important mechanism causing non-aflatoxigenicity in *A. flavus* populations. The current work suggests deletion of aflatoxin-biosynthesis genes is an ongoing process with genotypes retaining both varying percentages of the aflatoxin cluster and multiple genetic defects sufficient to cause non-aflatoxigenicity. Shared deletion patterns (Table 2; Fig. 2) suggest different deletion events lead to non-aflatoxigenicity in founding genotypes for the various lineages. A general pattern among genotypes was high frequencies of deletions (small and large) at the telomeric end of the cluster. This is likely a reflection of the neighboring sub-telomeric region which has a high rate of genetic flux compared to other regions (Chang et al. 2005; Liti and Louis 2005; Maciaszczyk et al. 2004).

Unlike the genotypes with partial gene clusters where most deletions were predominantly >1-kb, genotypes with complete gene sets have several smaller deletions (<1-kb) (Table 2). The smaller deletions were dispersed throughout the cluster with *norB*, *cypA*, *pksA*, *aflR, verA and verB* having higher frequencies of deletions. It is not surprising that *pksA* and *aflR* are two of the six genes, which are present in only 49 % of the genotypes analyzed in this study (Table 3). High frequencies of smaller deletions and higher probabilities of deletions in early portions of the gene cluster suggest that these genes may have gone through a combined process of inactivation by small deletions followed by complete gene loss from large deletions. Portions of the gene cluster are not only evolving differently but also going through different processes of degeneration (e.g. deletion vs SNPs) further suggesting multiple events are driving the degeneration of aflatoxin gene clusters.

### Differential evolution of aflatoxin gene cluster

Deletions in the gene cluster may not be sufficient to explain the variation among non-aflatoxigenic genotypes and mechanisms of non-aflatoxigenicity since genotypes with complete gene sets have no large deletions. SNP formation is an important mechanism through which non-aflatoxigenicity forms (Ehrlich and Cotty 2004). Three patterns are apparent in SNP distribution. First, with the exception of few genes (*norB*, *nor*-*1*), SNPs are common (Fig. 3; Additional file 1: Figure S1). Second, SNPs are not distributed randomly among genotypes. Third, late genes in the aflatoxin cluster have elevated SNP density as compared to early genes (Fig. 3). Not surprisingly, SNPs are also more abundant in non-aflatoxigenic genotypes than toxigenic genotypes as expected if purifying selection is maintaining toxigenicity. The observed variation in mutation in early and late portions of the cluster suggests the possibility of a differential evolution. Results from the current study reveal multiple forces driving evolution of aflatoxin genes and diverse mechanisms for non-aflatoxigenicity. This adds to recent observations (Grubisha and Cotty 2015) that non-aflatoxigenic phenotypes can be highly stable and retained for long-periods.

The physical order of the genes in aflatoxin gene cluster is similar to the order of enzyme reactions catalyzed by their gene products (Roze et al. 2007; Trail et al. 1995). However, expression of genes required for early stages of aflatoxin biosynthesis is modulated differently than those from the later steps (Ehrlich et al. 1999; Roze et al. 2007; Schmidt-Heydt et al. 2009). In agreement with these findings, are the different rates of evolution between early and late portions of the cluster (Fig. 3). This may result from selection driving divergent changes across the cluster. This hypothesis is consistent with the observation that two genes (*cypA* and *ver*-*1*) in the late portion are under positive selection (Fig. 4). This suggests that in non-aflatoxigenic phenotypes, certain genes in the late portion of the cluster are evolving new functions allowed by relaxation of selection for aflatoxin production. At the same time, lack of functional constraints might have allowed deletion of early genes in many genotypes. Occurence of deletions and other mutations at different rates in different portions of the cluster results in a complex process of aflatoxin biosynthesis loss.

### Evolution of clustered genes

Genes for biosynthesis of secondary metabolites are typically clustered together (Ehrlich et al. 2005; Keller and Hohn 1997; Walton 2000; Yu et al. 2004b). Genes encoding aflatoxin pathway enzymes are highly coexpressed. However, strong correlation coefficients between genomic changes suggest that certain genes involved in early steps of aflatoxin biosynthesis and neighboring *pksA* (Table 4) are evolving together and separately from the remainder of the cluster. The three genes (*hexA*, *hexB* and *pksA*) in the *pksA* group are involved in the conversion of acetate to norsolorinic acid (*nor*-*1*) (Yu et al. 2004a). The *pksA* gene, which is divergently transcribed from a 1.5 kb intergenic region with *nor*-*1*, along with *hexA* and *hexB* is involved in the synthesis of a polyketide from the primary metabolite acetate (Brown et al. 1996). Similar to the *pksA* group, genes in *sugR* group are also evolving together. None of the genes in the sugar cluster are evolving with any of the aflatoxin genes suggesting differential evolution and adaptation of the two clusters (Table 4). Clusters of genes that work together to produce a product (e.g., a secondary metabolite) may show coordinated changes in evolutionary rates because of increased or decreased utilization of those genes over evolutionary time.

### Implications for biological control of aflatoxin

Phenotypic variation among *A. flavus* genotypes confers differential adaptation to hosts, soils, and environmental conditions (Garber and Cotty 2014; Mehl and Cotty 2013; Mehl et al. 2012). Biocontrol methods that decrease human exposure to aflatoxins by selection and application of native, well adapted, non-aflatoxigenic genotypes of *A. flavus* have been successfully used in diverse locations and on several crops (Atehnkeng et al. 2008, 2014; Cotty 1994; Cotty et al. 2007; Probst et al. 2011, 2014). This study provides insight into diversity of mechanisms through which non-aflatoxigenicity has evolved. Twenty-six of the thirty-five non-aflatoxigenic genotypes of *A. flavus* included in the current analyses are active ingredients in biopesticides developed for aflatoxin management. Ten of the genotypes are active ingredients in biopesticides already registered for use (AF36 in the USA for *A. flavus* AF36 and AF36 Prevail™; NRRL 21882 in the USA for afla-guard®, Ka16127, La3279, La3304, and Og0222 in Nigeria for Aflasafe, and C6-E, C8-F, E63-I, and R7-H in Kenya for Aflasafe KE01) in aflatoxin management programs. Sixteen genotypes (GO18-2, GO67-10, MO11-8, MO92-15, M102-11, M109-2, M110-7, M129-5, M2-7, M21-11, Ms14-19, Ss19-14 AT21-A, BA35-C, DO114-A, EC69-E) are active ingredients in different biopesticides at various stages of the development cycle. The current report is the first report of the mechanisms of non-aflatoxigenicity for all the included genotypes except AF36 (Ehrlich and Cotty 2004) and NRRL 21882 (Chang et al. 2005). Molecular characterization of mechanisms of non-aflatoxigenicity is sometimes required for full biopesticide registration. The rational for this may be to verify the specific nature of the failure to produce aflatoxins or as a required tool for assessing the stability of non-aflatoxigenicity (Ehrlich and Cotty 2004; Grubisha and Cotty 2015). Sequences for aflatoxin biosynthesis gene clusters associated with the current report are also available to support tools for monitoring proportions of the specific non-aflatoxigenic genotypes in fungal populations associated with crops or in the environment, as well as, incidences of the specific mechanisms of non-aflatoxigenicity (Das et al. 2008; Mehl and Cotty 2010, 2013). Ten of the non-aflatoxigenic genotypes analyzed here are already being applied widely to commercial crops in the target nations (USA, Nigeria, or Kenya) and another sixteen are being applied to farmer’s fields on a smaller scale during biopesticide development in Senegal, Burkina Faso, and the USA. Thus, needs for the reported sequences already exist.

The structure of the aflatoxin biosynthesis cluster differs significantly among non-aflatoxigenic genotypes (Fig. 1). Closely related genotypes belonging to different VCGs have identical deletion patterns suggesting clonal derivation from a common ancestor. Deletion patterns within each of the 6 groups (group A to F; Tables 1, 2; Fig. 1) likely formed before separation into different VCGs. Multiple deletions in the aflatoxin gene cluster along with high frequencies of other mutations indicate origins of non-aflatoxigenicity in many lineages are old, sufficiently old to have allowed time, in some genes, for divergent adaptive evolution. In natural systems, multiple and diverse mechanisms of non-aflatoxigenicity in individual non-aflatoxigenic genotypes indicate long-term persistence and stability of non-aflatoxigenicity that extends over many thousands of years (Grubisha and Cotty 2010, 2015). In each of the non-aflatoxigenic gene clusters examined in the current study, once established, cluster non-aflatoxigenicity was maintained over sufficiently long periods for additional degeneration in the aflatoxin gene cluster to form. The multiple mechanisms of non-aflatoxigenicity suggest the clusters are not vulnerable to conversion to aflatoxin-producing phenotype and the *A. flavus* genotypes harboring these clusters cannot convert to aflatoxin-producers short of acquisition of an intact aflatoxin-biosynthesis gene cluster through either horizontal gene transfer or independent assortment of chromosomes during meiosis. Loci on all 8 chromosomes of *A. flavus* are in linkage disequilibrium with each other indicating that independent assortment of chromosomes does not occur among either aflatoxin producers (Grubisha and Cotty 2010) or among mixed populations of aflatoxin-producing and non-aflatoxigenic *A. flavus* (Grubisha and Cotty 2010, 2015; Ortega-Beltran et al. 2016). Although, simple mutations can cause non-aflatoxigenicity at any time in otherwise aflatoxin-producing lineages and such non-aflatoxigenics may revert back to aflatoxin-producer with a similar mutation, this is not the case for the non-aflatoxigenic genotypes used as active ingredients in biocontrol products. Non-aflatoxigenic active ingredients are selected from VCGs and/or SSR haplotypes that are relatively common and widely distributed in the target agroecosystem and which contain no aflatoxin-producing members (Atehnkeng et al. 2014; Bandyopadhyay and Cotty 2013; Cotty 2006; Probst et al. 2011). As revealed in the current report, these genotypes have numerous lesions in the aflatoxin biosynthesis gene cluster that have formed over long periods of non-aflatoxigenicity and are not vulnerable to conversion to aflatoxin-producing phenotype.

## Additional file

10.1186/s13568-016-0228-6 Validation of small deletions in aflatoxin gene clusters. **Table S2**. Validation of SNPs from aflatoxin biosynthesis gene clusters. **Table S3**. Annotation of SNPs from aflatoxin biosynthesis gene clusters. **Figure S1**. Heat map of SNP density in partial sets of genes in aflatoxin biosynthesis gene clusters.

## Abbreviations

SNP: Single Nucleotide Polymorphism
VCG: Vegetative Compatibility Group
USDA: United States Department of Agriculture
ARS: Agricultural Research Service
AGI: Arizona Genomics Institute
SSR: Simple Sequence Repeat
USEPA: United States Environmental Protection Agency
ANOVA: Analysis of Variance
