# Supplementary material for: Degeneration of aflatoxin gene clusters in Aspergillus flavus from Africa and North America
Source: AMB Express. 2016 Aug 31;6(1):62. doi: 10.1186/s13568-016-0228-6 (PMC5005231; doi:10.1186/s13568-016-0228-6)

Supplementary Material

Article Title: Degeneration of aflatoxin gene clusters in *Aspergillus flavus* from Africa and North America.
Journal name: AMB Express
Authors: Bishwo N. Adhikari, Ranajit Bandhyopadhyay, Peter J. Cotty
Affiliation: USDA-ARS, The University of Arizona, School of Plant Sciences, Tucson, AZ 85721
Email: pjcotty@email.arizona.edu

Table S1. Validation of small deletions (<1-kb) in aflatoxin gene cluster. Deletion validation was done by PCR amplification and sequencing of regions flanking deletion.

Genotype	*aflT* (253 bp)*	*pksA* (61 bp)	*hexA* (278 bp)	*aflR* (29 bp)	*verB* (57 bp)	*cypX* (21 bp)
AF36		Deletion§				Deletion
AT5-B						No deletion
BA16-F		Deletion		Deletion		Deletion
BY18-A	No deletion§		Deletion	Deletion		No deletion
C6-E	No deletion	Deletion	No deletion	Deletion	No deletion	Deletion
DO107-L	No deletion		No deletion	Deletion	No deletion	
EC69-E			No deletion	No deletion	No deletion	No deletion
Ka16127			Deletion			
La3304			Deletion	Deletion		
M011-8		Deletion	Deletion			Deletion
M129-5			Deletion	Deletion		
Ms14-19	Deletion	Deletion	No deletion		Deletion	Deletion
M102-11		Deletion		Deletion		No deletion

*Numbers in parenthesis indicate deletion size.
§Dark-shaded boxes indicate confirmed deletion (Deletion) and light-shaded boxes indicate confirmed absence of deletion (No deletion) by PCR amplification and sequencing of polymorphic regions.

Table S2. Validation of SNPs from aflatoxin biosynthesis gene cluster. SNP validation was done by PCR amplification and sequencing of the polymorphic sites.

Gene	Location	Location[a]	AF36	BY18-A	C6-E	MS14-19	OD02[b]
cypX	Late	Total	9	9	9	9	9
		Total correct	9	9	9	9	9
		Total incorrect	0	0	0	0	0
		% correct	100	100	100	100	100
estA	Early	Total	4	4	4	4	4
		Total correct	4	4	4	4	4
		Total incorrect	0	0	0	0	0
		% correct	100	100	100	100	100
moxY	Late	Total	4	4	4	4	4
		Total correct	4	4	4	4	4
		Total incorrect	0	0	0	0	0
		% correct	100	100	100	100	100
norA	Early	Total	5	4	4	5	5
		Total correct	5	4	4	5	5
		Total incorrect	0	0	0	0	0
		% correct	100	100	100	100	100
omtA	Late	Total	5	3	5	3	5
		Total correct	5	3	5	3	5
		Total incorrect	0	0	0	0	0
		% correct	100	100	100	100	100
pksA	Early	Total	5	6	5	5	6
		Total correct	5	6	5	5	6
		Total incorrect	0	0	0	0	0
		% correct	100	100	100	100	100

[a]Classification is based on the enzymes encoded by these genes, which are involved in early and late portions of aflatoxin biosynthesis, considering *ver-1* (shaded grey) as the middle gene.

[b]One of he three toxigenic isolates used in the analysis.

Table S3. Annotation of SNPs from aflatoxin gene cluster of 26 *Aspergillus flavus* genotypes.

Genotype	Type of SNP[∞]	*norB*	*cypA*	*aflT*	*pksA*	*nor-1*	*hexA*	*hexB*	*aflR*	*aflJ*	*adhA*	*estA*	*norA*	*ver-1*	*verA*	*avnA*	*verB*	*avfA*	*omtB*	*omtA*	*ordA*	*vbs*	*cypX*	*moxY*	*ordB*	*hypA*
AF36	SYN	0*	1	6	6	0	5	9	1	2	1	2	2	1	37	4	0	0	1	3	4	12	8	2	2	2
	NSYN	0	1	4	12	0	2	7	1	2	3	2	0	0	19	3	1	0	2	1	1	4	1	2	2	6
	Nonsense	§	¶	0	¶	0	0	0	0	0	0	0	0	0	0	0	0	0	0	0	0	0	0	0	0	0
BA16-F	SYN	0	3	5	18	1	18	20	3	9	2	6	5	3	5	4	5	4	1	7	6	10	6	7	1	2
	NSYN	0	7	1	16	1	15	10	3	2	4	2	3	1	5	4	4	4	2	3	3	6	3	6	1	2
	Nonsense	0	¶	0	0	0	⌘	0	0	0	0	0	¶	0	0	0	0	0	0	0	0	0	0	0	§	0
BY18-A	SYN	0	4	2	5	0	5	12	1	3	2	5	6	1	5	6	1	1	1	1	1	13	15	2	1	2
	NSYN	0	8	4	9	0	4	7	2	2	2	0	3	2	2	5	4	2	3	2	3	7	4	2	1	1
	Nonsense	§	¶	0	0	0	0	0	0	0	0	0	0	0	0	0	0	0	0	0	0	0	0	0	¶	0
C6-E	SYN	0	3	3	5	0	6	13	1	1	2	1	0	0	3	5	0	0	1	6	8	9	12	2	2	3
	NSYN	1	6	1	7	1	4	4	1	2	2	1	0	1	2	3	1	1	2	4	3	5	1	2	1	6
	Nonsense	§	§ ¶	0	0	¶	0	0	0	¶	0	0	0	0	0	0	0	0	0	0	0	0	0	0	0	0
DO107-L	SYN	0	3	3	2	0	1	5	2	2	2	6	10	2	2	5	1	2	1	1	3	10	12	3	0	3
	NSYN	1	5	1	1	1	1	3	1	2	1	0	3	1	1	4	4	1	1	1	3	5	3	2	1	5
	Nonsense	§	§ ¶	0	0	0	0	0	0	0	0	0	0	0	0	0	0	0	0	0	0	0	0	0	0	0
EC69-E	SYN	0	3	2	6	1	8	6	2	3	3	5	8	0	0	5	3	3	1	6	3	8	8	6	0	2
	NSYN	1	7	1	7	0	3	6	2	2	3	0	4	0	0	3	4	3	2	3	3	4	2	7	1	2
	Nonsense	§	§ ¶	0	0	0	0	0	0	0	0	0	0	0	0	0	0	0	0	0	0	0	0	0	0	0
GO67-10	SYN	0	2	2	1	0	2	4	0	6	2	5	9	1	3	5	1	2	0	1	0	10	9	4	0	1
	NSYN	1	4	1	3	0	0	0	1	2	1	1	3	0	1	4	3	1	1	3	1	5	2	4	1	2
	Nonsense	§	§ ¶	0	0	0	0	0	0	0	0	0	0	0	0	0	0	0	0	0	0	0	0	0	0	0
Ka16127	SYN	0	2	3	4	0	5	11	2	2	2	3	7	1	2	6	1	1	0	1	3	6	7	3	0	2
	NSYN	1	4	1	9	0	3	7	1	2	1	0	3	1	1	3	4	1	1	1	1	4	2	2	1	7
	Nonsense	§	§ ¶	0	0	0	0	0	0	0	0	0	0	0	0	0	0	0	0	0	0	0	0	0	0	0
La3279	SYN	0	3	5	4	1	7	13	1	2	1	2	4	2	2	4	1	1	0	3	1	2	3	1	1	1
	NSYN	1	4	2	8	1	2	5	1	3	1	0	2	1	1	3	4	1	1	1	0	4	1	1	1	2
	Nonsense	§	§ ¶	0	0	0	0	0	0	0	0	0	0	0	0	0	0	0	0	0	0	0	0	0	0	0
La3304	SYN	0	3	3	5	0	6	12	2	2	2	3	4	1	2	6	1	1	0	1	0	6	7	2	1	2
	NSYN	1	5	1	9	1	3	6	1	2	1	0	2	1	1	5	4	1	1	3	1	6	3	2	1	1
	Nonsense	§	§ ¶	0	0	0	0	0	0	0	0	0	0	0	0	0	0	0	0	0	0	0	0	0	¶	0
M011-8	SYN	0	3	4	5	0	7	13	2	2	2	4	9	1	2	6	1	2	1	5	6	7	12	3	1	3
	NSYN	1	6	2	9	1	2	5	1	2	1	0	3	1	2	3	4	1	1	2	3	3	1	3	1	7
	Nonsense	§	§ ¶	0	0	0	0	0	0	0	0	0	0	0	0	0	0	0	0	0	0	0	0	0	0	0
M102-11	SYN	0	2	2	5	0	7	13	2	2	2	2	0	1	2	5	2	1	0	2	1	10	9	2	0	2
	NSYN	1	5	2	7	1	2	5	1	1	2	0	1	0	1	4	4	1	1	2	2	6	2	1	1	1
	Nonsense	§	§ ¶	0	0	0	0	0	0	0	0	0	0	0	0	0	0	0	0	¶	0	0	0	0	0	0
M109-2	SYN	0	3	4	6	0	5	12	2	2	2	5	10	1	3	6	1	2	1	1	4	10	13	3	1	2
	NSYN	1	7	2	8	1	3	6	1	2	1	0	3	1	2	3	4	1	1	1	2	7	3	2	1	7
	Nonsense	§	§ ¶	0	0	0	0	0	0	0	0	0	0	0	0	0	0	0	0	0	0	0	0	0	0	0
M129-5	SYN	0	5	4	5	0	7	12	2	2	2	5	5	1	2	6	1	2	1	1	1	11	12	2	1	2
	NSYN	1	7	2	9	1	2	6	1	2	1	0	3	1	1	5	4	1	1	3	1	5	4	2	1	1
	Nonsense	§	§ ¶	0	0	0	0	0	0	0	0	0	0	0	0	0	0	0	0	0	0	0	0	0	¶	0
M2-7	SYN	0	2	3	6	0	6	11	2	2	2	4	8	1	3	5	1	1	0	1	0	8	7	2	1	2
	NSYN	1	6	1	8	1	2	6	1	2	1	0	3	1	1	3	3	1	1	3	1	6	4	2	1	1
	Nonsense	§	§ ¶	0	0	0	0	0	0	0	0	0	0	0	0	0	0	0	0	0	0	0	0	0	¶	0
Ms14-19	SYN	0	3	3	13	0	20	19	4	9	4	6	6	1	6	5	3	4	2	6	4	10	7	7	0	5
	NSYN	1	8	2	15	1	10	9	4	3	3	2	2	2	3	3	5	4	1	4	3	4	2	6	1	3
	Nonsense	§	§ ¶	0	0	0	0	0	0	0	0	0	0	0	0	0	0	0	0	0	0	0	0	0	¶	0
Ss19-14	SYN	0	5	3	16	0	19	18	4	9	4	4	6	2	6	5	4	4	2	6	5	10	16	1	1	4

	NSYN	1	6	2	12	1	11	10	4	3	3	4	2	2	3	3	6	4	2	3	3	6	4	1	1	3
	Nonsense	§	§ ¶	0	0	0	0	0	0	0	0	0	0	0	0	0	0	0	0	0	0	0	0	0	¶	0
C8-F	SYN	1	3												1	5	3	1	1	3	9	2	6	7	0	4
	NSYN	0	6												1	2	3	1	3	1	5	1	3	6	0	3
	Nonsense	0	¶												0	0	0	0	0	0	0	1	0	0	§	0
DO38-B	SYN	0	3												0	5	2	3	1	7	4	8	8	7	1	2
	NSYN	0	7												1	2	2	3	3	4	3	3	2	8	1	1
	Nonsense	0	¶												0	0	0	0	0	0	0	0	0	0	§	0
R7-H	SYN									0	2	1	0	0	0	4	2	0	0	5	2	9	9	2	1	2
	NSYN									1	2	1	0	1	1	2	1	0	0	3	2	3	3	2	1	2
	Nonsense									0	0	0	0	0	0	0	0	0	0	0	0	0	0	0	0	0
E63-I	SYN									1	2	2	0	0	0	4	5	0	3	7	4	10	11	2	1	2
	NSYN									1	1	1	0	1	1	2	1	0	1	4	3	6	3	2	1	2
	Nonsense									0	0	0	0	0	0	0	0	0	0	0	0	0	0	0	0	0
M21-11	SYN																		0	10	2	10	10	2	2	2
	NSYN																		0	4	2	6	4	2	2	2
	Nonsense																		0	0	0	0	0	0	0	0
AT5-B	SYN													3	2	2	5	4	2	6	5	12	5	7	0	5
	NSYN													1	1	5	3	4	3	4	2	4	3	6	1	3
	Nonsense													0	0	0	0	0	0	0	0	0	0	0	0	¶
DO114-A	SYN													1	2	4	4	4	2	0	4	10	7	6	1	5
	NSYN													1	1	3	4	4	2	0	4	9	3	6	1	3
	Nonsense													0	0	0	0	0	0	0	0	0	0	0	§	¶
M092-15	SYN													1	2	3	4	5	1	6	6	10	7	7	0	3
	NSYN													1	1	4	4	4	2	4	2	6	2	6	1	3
	Nonsense													0	0	0	0	0	0	0	0	0	0	0	0	¶
GO18-2	SYN													0	0	2	5	4	1	6	4	11	5	5	1	2
	NSYN													0	1	5	3	4	3	3	2	5	2	4	1	1
	Nonsense													0	0	0	0	0	0	0	0	0	0	0	§	¶

∞SYN, Synonymous SNP, NSYN, Non-synonymous SNP.

*Numbers indicate SNP count. Boxes are shaded gray where part of the aflatoxin gene cluster is deleted.

§Indicates loss of at least one Stop codon.

¶Indicates gain of at least one Stop codon or nonsense SNP.

⌘Indicates loss of at least one Start codon.

Fig. S1 Heat map of SNP density (SNPs/kb of gene) in partial sets of genes in aflatoxin biosynthesis cluster of 9 non-aflatoxigenic *A. flavus* genotypes. SNPs were called in reference to *A. flavus* AF13. Genes with a common letter at the bottom do not differ significantly in mean SNP density by Tukey's HSD test ($P<0.05$) performed in genes present in all 9 genotypes. Schematic representation of the aflatoxin biosynthesis cluster is presented at the top. Left is the telomeric end of the cluster.

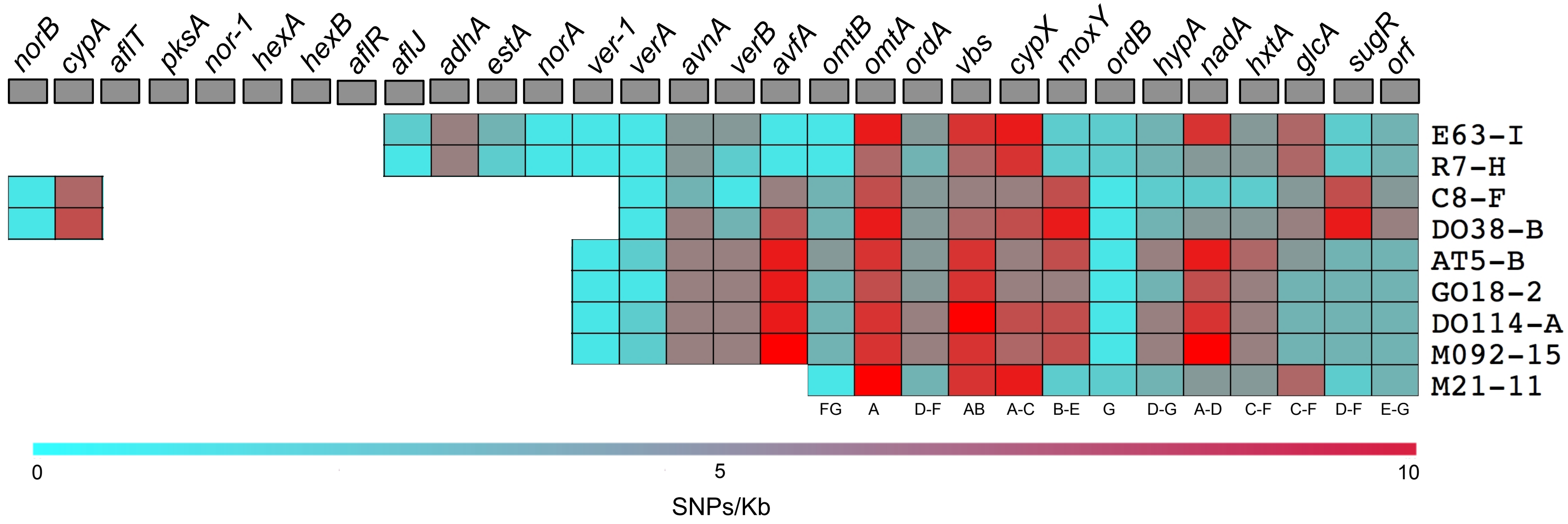

Supplement: Supplementary file 1 — 10.1186/s13568-016-0228-6 Validation of small deletions in aflatoxin gene clusters. Table S2. Validation of SNPs from aflatoxin biosynthesis gene clusters. Table S3. Annotation of SNPs from aflatoxin biosynthesis gene clusters. Figure S1. Heat map of SNP density in partial sets of genes in aflatoxin biosynthesis gene clusters. [file 13568_2016_228_MOESM1_ESM.pdf]
